# Crossmodal interactions in human learning and memory

**DOI:** 10.3389/fnhum.2023.1181760

**Published:** 2023-05-17

**Authors:** Carolyn A. Murray, Ladan Shams

**Affiliations:** ^1^Department of Psychology, University of California, Los Angeles, Los Angeles, CA, United States; ^2^Department of Bioengineering, Neuroscience Interdepartmental Program, University of California, Los Angeles, Los Angeles, CA, United States

**Keywords:** multisensory, perceptual learning, adaptation, recalibration, multisensory memory, multisensory learning

## Abstract

Most studies of memory and perceptual learning in humans have employed unisensory settings to simplify the study paradigm. However, in daily life we are often surrounded by complex and cluttered scenes made up of many objects and sources of sensory stimulation. Our experiences are, therefore, highly multisensory both when passively observing the world and when acting and navigating. We argue that human learning and memory systems are evolved to operate under these multisensory and dynamic conditions. The nervous system exploits the rich array of sensory inputs in this process, is sensitive to the relationship between the sensory inputs, and continuously updates sensory representations, and encodes memory traces based on the relationship between the senses. We review some recent findings that demonstrate a range of human learning and memory phenomena in which the interactions between visual and auditory modalities play an important role, and suggest possible neural mechanisms that can underlie some surprising recent findings. We outline open questions as well as directions of future research to unravel human perceptual learning and memory.

## 1. Introduction

The environment and set of tasks the human brain must complete throughout the course of our lives create an immense challenge for the nervous system. We live in dynamic environments, whose changes require a large variety of flexible behaviors to navigate. Moreover, the human body also changes through time, growing when we are young and deteriorating with age. The brain must recalibrate and adjust its functioning during all of these stages in life. The complexity of these systems is such that it is not possible for all behaviors to be hard-coded; the human genome only contains 20–25 thousand genes, which is far too few to code everything the brain must compute and perform. In addition, humans are social animals, which will require us to not just have a functional understanding of our physical environment, but of our social experiences and networks as well.

These complex environmental and developmental factors have thus necessitated the evolution of a brain that is capable of recalibration and learning. The human brain is, in fact, noted for being incredibly plastic (Kolb and Whishaw, 1998; Calford, 2002), and apt at both supervised and unsupervised learning (Knudsen, 1994). In addition, the human brain is accomplished in memory tasks that support learning about our environments and remembering our social interactions. As they are such fundamental functions of human behavior, both learning and memory have been studied extensively in humans over the decades in a variety of disciplines and using a variety of methods. However, the vast majority of these studies focus on studying one sense at a time [for overviews, see Goldstone (1998), Fiser and Lengyel (2022)].

While situations that focus on the experiences of only one sense can be created in an experimental space, such work does not reflect the cues across many senses that would be available and working in concert in a natural environment. On a daily basis, we use information across multiple senses to learn about our environment and encode in our memories for later use. The senses do not operate in a vacuum. If we drop a glass, we do not just see it fall, but we hear the impact and feel the lack of its weight in our hands. When talking to a friend, we do not just hear their voice, but see their facial expressions and smell their perfume. With such rich information available across senses about the same experience, it would make sense if the brain was capable of processing this information in a holistic way, without the boundaries of sensory modality and perhaps even exploiting the relationship between the sensory cues. Yet, the vast majority of studies of perceptual learning and memory have used unisensory stimuli and tasks.

Research over the last two decades, however, has greatly enhanced our understanding of how the brain is able to combine information across the senses. Myriad studies have established that sensory pathways can influence one another, even at their earliest stages. For example, the presence of low-level multisensory illusions, such as the ventriloquist illusion (Thurlow and Jack, 1973; Bruns, 2019) and the sound-induced flash illusion (Shams et al., 2000; Hirst et al., 2020) indicate that the senses combine information early on and influence one another in ways that are observable at a behavioral level. Psychophysical studies have established that the interactions between the senses is ubiquitous, they occur across all sensory modalities and many tasks (e.g., Botvinick and Cohen, 1998; Shams et al., 2000; Wozny et al., 2008; Peters et al., 2015; Bruns, 2019), and across the lifespan (e.g., Setti et al., 2011; Burr and Gori, 2012; Nardini and Cowie, 2012; Murray et al., 2016a; McGovern et al., 2022). Accordingly, brain studies have revealed interactions between the senses at a variety of processing stages, in all processing domains (Murray et al., 2016b; Ferraro et al., 2020; Gau et al., 2020, and see Ghazanfar and Schroeder, 2006; Driver and Noesselt, 2008; for reviews). Altogether, research has uncovered that multisensory processing is not simply the sum of unisensory processes, which implies that multisensory learning cannot be simplified to the sum of the constituent unisensory learning and memory. Indeed, researchers have begun investigating learning and memory under multisensory conditions, and these studies have revealed surprising phenomena that point to multisensory processing being a unique and powerful mechanism for learning and memory.

Here, we will briefly review some of the studies that investigate learning and memory through a multisensory lens, with a particular focus on audio-visual studies. We will additionally focus on studies performed in healthy human adults, though there is significant work studying multisensory learning during development (e.g. Gori et al., 2008; Nardini and Cowie, 2012; Dionne-Dostie et al., 2015; Murray et al., 2016a), in clinical populations (e.g., Held et al., 2011; Landry et al., 2013; Stevenson et al., 2017), and in animals (e.g., Wallace et al., 2004; Xu et al., 2014). We will highlight key takeaways from healthy human adult research as a whole. Building upon neural mechanisms proposed by Shams and Seitz (2008), we will outline possible neural mechanisms that may explain the relative potency of multisensory learning/memory when compared to unisensory variations, and a larger range of learning phenomena including some surprising recent behavioral findings. We additionally suggest directions for future research.

## 2. Multisensory learning

The topic of multisensory learning has been broadly approached under a number of labels, including but not limited to studies of multimedia learning (Mayer, 2014) or Montessori education (Montessori, 2013). However, many of these studies, by nature of being more applied in nature, are often not rigorous experiments with appropriate controls. Thus, the results are frequently not easy to interpret. In our discussion of multisensory learning, we will focus on experimental studies that, in addition to using rigorous experimental methods, also shed light on underlying mechanisms that could explain multisensory benefits. These studies have tackled a variety of learning ranging from supervised perceptual learning to unsupervised or implicit types of learning such as recalibration and adaptation.

### 2.1. Perceptual learning

Perceptual learning can be defined as a refinement in perceptual processes, improving detection and discrimination of stimuli through perceptual experience (Gold and Watanabe, 2010). Because the experience is crucial for improvement, there has been significant interest in developing training regimens that will support perceptual learning. Sensory training has been long studied in unisensory contexts (for examples, see reviews by Goldstone, 1998; Fiser and Lengyel, 2022). However, studies in multisensory perceptual learning have emerged in the past two decades that indicate this learning is not solely a unisensory phenomenon, and that multisensory training has the potential to be a powerful tool for refining perception above and beyond that obtained by unisensory training.

One fascinating benefit of multisensory training is the ability for this sensory information to refine not just multisensory processing, but to improve on unisensory processing. In the domain of motion processing, audio-visual training has been shown to be superior to visual training both in the overall degree of learning as well as rate of learning, even when compared on trials consisting only of visual information (Seitz et al., 2006). Furthermore, a later study (Kim et al., 2008) showed that the congruence between the auditory and the visual motion during training was necessary for this multisensory training benefit. Training with incongruent audiovisual stimuli did not lead to improved learning compared to visual-alone training, even though the stimuli in the incongruent condition were equally arousing as those in the congruent condition. These results suggest that integration of auditory-visual stimuli is critical for the facilitation and enhancement of learning, making the benefit a matter of multisensory mechanisms being used, rather than a mere effect of heightened neural activity due to potentially increased arousal.

**FIGURE 1:**
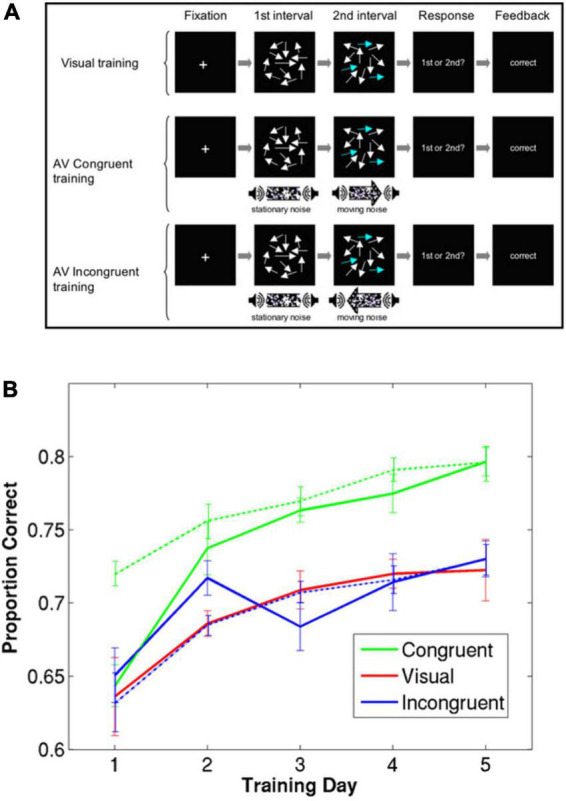
Perceptual learning of motion coherence detection in three different training conditions, adapted from Kim et al. (2008). **(A)** Multisensory training and visual training and test conditions presented to participants in Kim et al. (2008). **(B)** A group of participants was trained using only visual stimuli (red), a group was trained using visual and auditory stimuli moving in the same direction (green), and a group was trained using visual and auditory stimuli moving in opposite directions (blue). A fraction of trials in the multisensory training groups were unisensory (visual only). The solid lines and broken lines represent performance on visual-only trials and audiovisual trials, respectively. Error bars represent within-group standard errors (*n* = 7).

In this study, the participants in the multisensory training groups were trained with sessions that consisted of mostly auditory-visual trials, however, it also included some visual-only trials. This design also allowed comparing the accuracy in unisensory versus multisensory trials for each subject throughout training. Figure 1 shows the detection accuracy for the congruent auditory-visual training group for both auditory-visual trials (broken green line) and visual-alone trials (solid green line). In auditory-visual trials, there is task-relevant information (i.e., which of the two intervals contains coherent motion) in both modalities, whereas in the visual-alone trials that information is only available in the visual modality. The coherence level of visual stimuli were equivalent between visual-alone and auditory-visual trials. Therefore, it was expected that performance in auditory-visual trials to be higher than that of visual-only trials. Indeed, in the early training sessions, participants’ performance was higher in the audiovisual trials than in visual trials. However, this difference decreased over subsequent training sessions, and finally the performance in the visual-only trials matched that of auditory-visual trials by the end of training (Figure 1). This intriguing finding has important implications for unraveling the computational mechanisms of multisensory learning as we discuss later.

Work by von Kriegstein and Giraud (2006) showed that neural changes that occurred during multisensory learning could explain such phenomena. Training individuals on audiovisual voice-face associations strengthened the functional connection between face- and voice-recognition regions of the brain. They argue that this means that multisensory training has the means to improve unisensory perceptual improvement because later unisensory representations have the ability to activate larger ensembles due to increased connectivity through multisensory training. To that end, multisensory training has the ability to be more effective for perceptual learning than unisensory alternatives, perhaps as a result of multisensory mechanisms that will be discussed in more depth in the Neural Mechanisms section below.

In a more recent study, Barakat et al. (2015), investigated the multisensory training benefit in the context of rhythm perception. Participants were asked to make same/different judgments on visual rhythms. Participants were trained in either a visual only condition, an auditory only condition, or a multisensory condition, where identical auditory and visual rhythms were played simultaneously. In line with previous findings, but even more strikingly, they found that participants who underwent the multisensory training improved in the visual task substantially and already after one training session, in contrast to the participants who underwent visual-only training who showed no significant improvement even after two training sessions. Perhaps more surprising, however, was the finding that the auditory training was as effective as multisensory training, even though sound was completely absent in the test task. This pattern of results suggests that the visual and auditory regions must be communicating with one another even in the absence of a multisensory training, meaning crossmodal mechanisms must be engaged even in the absence of direct stimulation.

These findings are consistent with those of a more recent study that examined crossmodal transfer of learning in both spatial and temporal tasks in both vision and hearing (McGovern et al., 2016). The results showed that in a given task training in sensory modality that is relatively more accurate (e.g., vision in a spatial task, hearing in a temporal task) leads to improved performance of the less accurate sensory modality in the same task. Such findings cannot be easily explained by traditional theories of perceptual learning. Possible neural mechanisms mediating these phenomena will be further explored later in the (section “4. Neural mechanisms”).

While the aforementioned studies have trained observers on performing a perceptual task that can be done using both unisensory and multisensory stimuli (e.g., detecting motion), other studies have investigated the effect of training observers on a task involving determination of the temporal relationship between crossmodal stimuli, namely, the simultaneity or the temporal order of two crossmodal stimuli (e.g., Powers et al., 2009; Alais and Cass, 2010; see O’Brien et al., 2023 for a recent review). These studies have reported improved performance on the trained tasks (e.g., Virsu et al., 2008; Powers et al., 2009; Alais and Cass, 2010; De Niear et al., 2018), and in some cases also a transfer of learning to other tasks involving crossmodal stimuli (e.g., Setti et al., 2014; McGovern et al., 2016; Powers et al., 2016; Sürig et al., 2018, but see Horsfall et al., 2021; O’Brien et al., 2020). These findings demonstrate the fast plasticity of the perceptual processes even at foundational level of time representation. However, the exact mechanism underlying the improved performance (i.e., narrowing of the time window of simultaneity or improved temporal acuity) requires further research. Improved performance in these tasks could be due to either the improved unisensory temporal precision, or a modification of multisensory mechanisms, or both. Future research can elucidate this by testing observers in unisensory tasks before and after training, and/or using the Bayesian Causal Inference model to quantitatively probe the unisensory precisions as well as multisensory processing components before and after training.

### 2.2. Recalibration

While perceptual learning studies typically involve giving feedback to the participants about the accuracy of their responses, and therefore are a form of supervised learning, other types of learning that occur naturally in nature and do not involve explicit feedback also play an important role in being able to function in an ever-changing environment. For example, the brain needs to be able to maintain coherence of information across the senses. Were the senses truly independent, it wouldn’t be possible to use one to calibrate another. Thus, crossmodal interactions are also critical in maintaining the accuracy of sensory measurements and representations in face of environmental and bodily changes. It is well established that the human nervous system is capable of recalibrating the sensory systems even in maturity in various processing domains (e.g., Recanzone, 1998; Fujisaki et al., 2004; Vroomen et al., 2004). For example, repeated exposure to auditory-visual stimuli with a fixed spatial discrepancy leads to a subsequent shift in the map of auditory space in the direction of the previously experienced visual stimuli, in a phenomenon known as the *ventriloquist aftereffect* (Recanzone, 1998). This is a clear illustration of the use of the visual input as a teaching signal to calibrate the auditory representations. Indeed, quantitatively modeling the observer’s localization responses before and after exposure to spatially discrepant auditory-visual stimuli has shown that it is the sensory (namely, auditory) representations that are shifted in ventriloquist aftereffect rather than a prior expectation of stimuli or a combination of the two (Wozny and Shams, 2011).

While earlier studies had utilized extended exposure (hundreds or thousands of trials, or minutes or hours of exposure), a more recent study (Wozny and Shams, 2011) showed that long exposure is not required to trigger and engage the recalibration process. A single exposure lasting only a fraction of a second to a spatially discrepant audiovisual stimulus can cause a shift in spatial localization of an ensuing auditory stimulus presented alone (Wozny and Shams, 2011). Recalibration in the span of a fraction of a second indicates that the nervous system is extremely sensitive to discrepancy across senses and seeks to resolve it expeditiously. Because multisensory stimuli can be used in such rapid recalibration, they are uniquely poised as crucial to help the brain to keep up with a dynamic environment. The effects of recalibration can be long-lasting, to match the environment; for example, multisensory recalibration in the ventriloquist aftereffect has been shown to persist over the course of days, with appropriate training (Bruns, 2019).

While recalibration has been studied extensively both at a behavioral and neural level in both humans and animal models (for example, Knudsen and Knudsen, 1985; Wallace et al., 1998; Kopco et al., 2009; Aller et al., 2022) the computational characterization of this process had not been investigated systematically until recently. Wozny and Shams (2011) probed the role of causal inference in the visual recalibration of auditory space in the same study. Recalibration seemed significantly stronger on trials where observers appeared to have inferred a common cause for the auditory and visual stimuli compared to those where did not appear to perceive unity. Auditory recalibration by vision also appears to be better explained by Bayesian Causal Inference than by competing models of sensory reliability or fixed-ratio recalibration (Hong et al., 2021). Such findings are surprising because recalibration is traditionally considered a very low-level phenomenon, occurring at early stages of sensory processing [as in Zwiers et al. (2003); Fujisaki et al. (2004)], whereas causal inference is considered a high-level process, occurring in later stages of cortical processing (Kayser and Shams, 2015; Rohe and Noppeney, 2015; Aller and Noppeney, 2019; Cao et al., 2019; Rohe et al., 2019; Ferrari and Noppeney, 2021). Recent works are challenging this distinction, however; it has been recently suggested that recalibration can be subject to top-down influences from higher cognitive processes (Kramer et al., 2020), and that regions involved in both perception and decision-making are flexibly involved in the recalibration process (Aller et al., 2022). Such findings support the computational evidence that low-level perceptual and higher-level computational processes may not be as distinct as originally theorized, and therefore, causal inference could influence the recalibration process.

### 2.3. Implicit associative learning

Implicit associative learning is another form of unsupervised learning, where a new association is learned based on passive exposure to statistical regularities of the environment (Reber, 1967; Knowlton et al., 1994; Saffran et al., 1996; Aslin, 2017; Batterink et al., 2019; Sherman et al., 2020). Observers are able to implicitly learn the association between crossmodal stimuli, even when the association is entirely arbitrary. For example, exposure to arbitrary association between visual brightness and haptic stiffness results in refined discrimination of visual brightness (Ernst, 2007).

Because this type of learning involves extraction of statistical regularities in the environment it falls under the umbrella of statistical learning, broadly speaking. Statistical learning has been studied often from a unisensory perspective (Conway and Christiansen, 2005), but studies that have examined statistical learning across sensory modalities have often reported a powerful and fast learning of links (joint or conditional probabilities) between the senses, such as shape and sound (Seitz et al., 2007). In a study that compared the rate of learning of within-modality regularities vs. across-modality regularities, it was found that observers learned auditory-visual regularities more effectively than visual-visual or auditory-auditory ones (Seitz et al., 2007). Therefore, it appears that the nervous system is particularly apt at detecting statistical relationships across the senses. However, there may be constraints on temporal relationships that lend themselves to learning of crossmodal statistical regularities. Many studies showing multisensory benefit in implicit association tasks utilize simultaneous audiovisual presentation, but some studies indicate that learning multisensory associations through time, including between color and tone (Conway and Christiansen, 2006) or crossmodal artificial grammar sequences (Walk and Conway, 2016) may be more challenging to learn than within-modality associations. Such findings potentially suggest there may be limitations to the types of procedures that will produce effective multisensory learning. Such suggestions do not preclude that multisensory learning is possible, just that the constraints on this learning may be different from those on unisensory learning (Frost et al., 2015). The necessity of crossmodal synchronicity for effective implicit associative multimodal learning is thus an open question in need of more research.

It should also be noted that, as with other forms of learning discussed earlier, benefits can be observed even when one modality present during learning is irrelevant at test. In a study in which participants were passively exposed to co-occurring visual and auditory features in the background, and in a subsequent visual test, they exhibited improved sensitivity to visual features in presence of the associated sound, even though the sound was task-irrelevant (Shams et al., 2011). Altogether, these findings highlight that multisensory encoding of information is able to improve unisensory representation and processing, even if the relationship between the two stimuli in different senses is arbitrary.

In fact, learning associations that are seemingly arbitrary could be a crucial step in learning meaningful associations. Learning of crossmodal correspondences– information across senses that are arbitrary yet are robustly considered “congruent”– are an important area of study within multisensory processing (for reviews, see Spence, 2011; Parise, 2016). Such correspondences have been studied across a wide variety of sensory pairs, including auditory timbre and visual properties such as shape and color (Adeli et al., 2014), haptic assessment of heaviness and auditory pitch (Walker et al., 2017), visual hue and tactile texture (Jraissati et al., 2016), and visual color and gustatory taste profile of an object (Spence et al., 2010). While these associations range from the seemingly sensible to the entirely arbitrary, they usually evolve from some type of association present in the environment to some extent (for discussion, see Parise, 2016), and thus reflect a great flexibility in crossmodal learning in order to map such seemingly arbitrary associations. While the crossmodal correspondence is rightly treated as related yet separate from a truly multisensory process, current research indicates that crossmodal correspondences, once learned, can influence multisensory integration. Training in an arbitrary but “congruent” crossmodal correspondences has been shown to prime later multisensory integration (Brunel et al., 2015), and as such may represent a crucial stage in understanding how the brain learns to integrate novel crossmodal pairs. The neural mechanisms by which such crossmodal correspondences develop and persist remain unclear; though it has been posited that they may be the same mechanisms that underlie the phenomenon of synesthesia (Parise and Spence, 2009), further research into the mechanisms investigating how crossmodal correspondences contribute to multisensory integration are required.

## 3. Multisensory memory

The benefits of multisensory processing are not limited to just the realm of learning. The memory systems of the brain must also, crucially, be able to store and represent information across senses in order for humans to make sense of our environment. In addition, our episodic memory, as well as being a useful guide on our environment, helps us to store information crucial to the events of our lives, which helps us to store information crucial to social interactions and aid in decision making critical for survival. Episodic memory is commonly defined as memories for events and experiences, rich in sensory and contextual details, rather than memories for facts (Tulving, 1993). Memories are rich in sensory detail and can typically be cured by many senses. Neuroimaging studies have revealed that the role of perception in memory was not unidirectional upon encoding: recall of visual and auditory stimuli reactivates sensory-specific cortices that were active at encoding. This is true within modality, where a sensory region active during encoding is reactivated upon recall (Nyberg et al., 2000) but has also been shown in multisensory conditions, where a visual probe for an audio visually-encoded item reactivates auditory regions as well as visual ones (Wheeler et al., 2000). This highlights a clear link between sensory representations and mnemonic codes. Many studies of human memory have focused on individual senses (for examples, see Weinberger, 2004; Brady et al., 2008; Slotnick et al., 2012; Schurgin, 2018) or chosen to not view memory through a sensory lens at all. However, given that multisensory training has now been shown to benefit learning (Shams and Seitz, 2008), and that episodic memory ties together information across senses in a way that seems to naturally take advantage of crossmodal processing, work in the past two decades has begun to explore the benefits of multisensory stimulus presentation for memory performance.

Research on object recognition has shown that multisensory presentation of objects during the encoding phase seems to enhance later recognition of unisensory representation of the objects. Recognition performance for visual objects presented initially with congruent audio and visual cues was reported to be higher than that of objects initially presented only visually, or with an incongruent audio (Lehmann and Murray, 2005; Thelen et al., 2015). When the recognition test is auditory instead of visual, the pattern of results has been shown to be similar, where multisensory encoding produces higher recognition than audio-alone encoding (Moran et al., 2013).

The aforementioned studies all used a continuous recognition task in which the first and second presentations of the same object are presented within a stream of objects that are interleaved. Experiments that use a more traditional memory paradigm, with distinct encoding and retrieval phases separated by a delay interval, and also those attempting to study more naturalistic tasks have also found a benefit to multisensory encoding. Heikkilä et al. (2015) used such a paradigm to compare benefits in visual recognition to benefits in auditory recognition for stimuli encoded in a multisensory condition compared to stimuli encoded in a unisensory fashion. Contrary to some earlier studies, this study found no benefit to visual recognition between the two conditions, though there was a significant improvement to recognition for auditory memory for items encoded with a visual compared to those encoded as audio only. This study also looked for improvement in recognition of spoken and written words and found that adding audio to written words and vice versa improved recognition, so the benefits seen in previous studies may not be limited to perceptual representations and appear to extend to semantic information. A recent study reported a weak but significant benefit of congruent auditory-visual encoding compared to unisensory or incongruent auditory-visual encoding, in auditory recognition but not in visual recognition (Pecher and Zeelenberg, 2022). In both of these studies, there is an asymmetry in the effect of multisensory encoding on recall: auditory representations benefit from multisensory training whereas visual representations do not. Given that auditory recognition memory is typically noted for being worse than its visual counterpart (Cohen et al., 2009; Gloede and Gregg, 2019), the representations supporting auditory memory may be more ambiguous, and thus may particularly benefit from multisensory encoding.

Findings supporting multisensory benefit to memory performance are not limited to recognition memory paradigms. A recent study showed that recall for visual objects was better when those objects were initially presented with congruent auditory information, even if participants were explicitly told to ignore that auditory information (Duarte et al., 2022). In a similar pattern of results, it was shown that recall of face-name associations could be bolstered by the addition of a name tag that was congruent with the auditory name presentation, extending findings of multisensory memory benefits to associative memory tasks (Murray et al., 2022). These behavioral findings are in line with previous fMRI results showing that higher activation in audiovisual association areas is observed during encoding for face-name pairs that will be later remembered compared with those that will be forgotten (Lee et al., 2017). On the whole, these findings suggest that multisensory encoding is a means by which memory retrieval can be improved, even in complex and naturalistic contexts.

## 4. Neural mechanisms

The benefits to perceptual learning, recalibration, adaptation, and memory mentioned thus far have largely been discussed in terms of behavioral studies. This leaves the question of what neural mechanisms may underpin the aforementioned findings and would explain the superiority of multisensory encoding over unisensory encoding/learning. This question remains somewhat open, with many proposed theories holding some weight from the multisensory literature.

**FIGURE 2:**
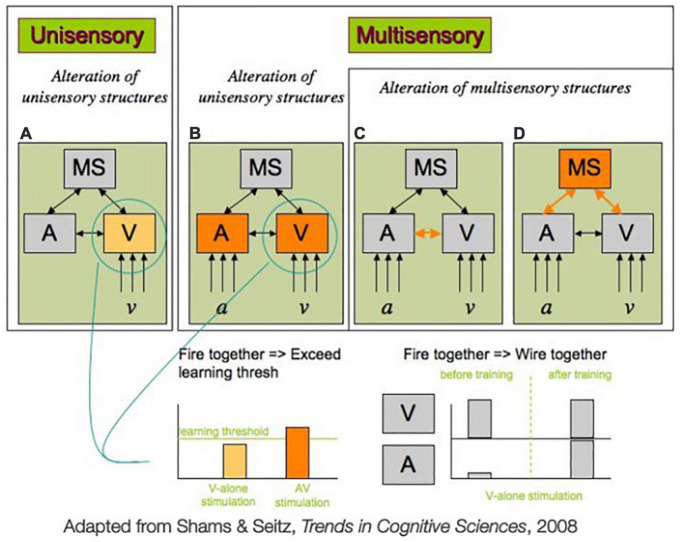
Two possible mechanisms mediating multisensory training advantage for unisensory processing, adapted from Shams and Seitz (2008). **(A)** In classic perceptual learning studies, only one sensory modality (e.g., vision) is trained. In such a unisensory training paradigm, learning would only modify the existing unisensory features (e.g., visual representations, *v*, or auditory representations, *a*, here). In multisensory training paradigms **(B–D)** multiple sensory modalities (e.g., vision and hearing) are stimulated simultaneously. The advantage of multisensory training over unisensory training could be due to **(B)** the fact that the pre-existing connection between the sensory regions (A and V, here) gives rise to a higher activity of each unisensory region (e.g., V) as compared to unisensory stimulation and exceeds the threshold required for learning to occur. Alternatively, multisensory training which involves repeated co-activation of unisensory regions A and V could result in strengthening of multisensory structures (MS here), such as direct connection between unisensory regions, as depicted in **(C)** or the connection between unisensory regions and multisensory regions, as depicted in **(D)**, or both in a “fire together, wire together” fashion. As a result of this new wiring, the activation of one unisensory region can lead to activation of the other unisensory region [either via direct connection **(C)** or indirectly through multisensory connections **(D)** or both], in effect implementing redintegration (see section “4. Neural mechanisms” for more detail).

Generally, theories fall into two categories: those that make the assumption that learning occurs with neural changes to unisensory regions, and those that make the assumption that learning reflects changes in multisensory structures or crossmodal connectivity (Figure 2; Shams and Seitz, 2008). In unisensory theories, the assumption is made that, through training, unisensory regions will eventually refine their processing. This occurs, in a unisensory context, when activity in a unisensory region is heightened above a learning threshold (Figure 2A). Under this framework, multisensory training encourages learning by making it easier to elevate the neural activity above the level of the learning threshold, because it activates neural populations both in the sense that is being targeted, and in another region corresponding to another sense that has crossmodal connections to the sense being targeted (Figure 2B). These crossmodal connections raise activity in the targeted region above what would be possible if it was stimulated in isolation, making it easier to surpass the learning threshold, and thus leading to faster learning in multisensory training conditions. Such a model could explain the findings that report multisensory encoding of objects does lead to distinct brain activation at retrieval that is not observed with unisensory encoding (Murray et al., 2004; Thelen and Murray, 2013).

By contrast, multisensory frameworks posit that learning is more in line with a Hebbian learning model, following the principle of “fire together, wire together” for the unisensory and multisensory regions (Hebb, 1949; Magee and Grienberger, 2020). Multisensory learning can occur during several different levels under this framework, but we will focus on the idea that plasticity occurs in either the connectivity between unisensory areas that are co-firing during multisensory training (Figure 2C) or multisensory regions and their connections to unisensory areas that are strengthened during co-firing (Figure 2D). Under either of these mechanisms, learning takes place in part because the two senses contributing to a multisensory signal are co-occurring, which encourages these regions to become more strongly connected. This stronger connection will allow for activation of one region to more easily recruit a larger population of neurons post-training, due to stronger crossmodal connections.

A recent review by Mathias and von Kriegstein (2023), focusing on neuroscience and neurostimulation in the area of multisensory learning came to the conclusion that multisensory mechanisms, consistent with those posited in Figures 2B–D, appear to be a better explanation for the observed benefits from multisensory learning as opposed to unisensory learning mechanisms (as would be consistent with those posited in Figure 2A). They report on imaging and neurostimulation studies that report that functional connectivity between sensory-specific areas is altered after crossmodal learning [as in von Kriegstein and Giraud (2006), Thelen et al. (2012), Mayer et al. (2015)]. It has also been suggested via simulation studies that both crossmodal connectivity and connections between unisensory regions and higher-level association areas could be strengthened simultaneously during multisensory learning (Cuppini et al., 2017).

**FIGURE 3:**
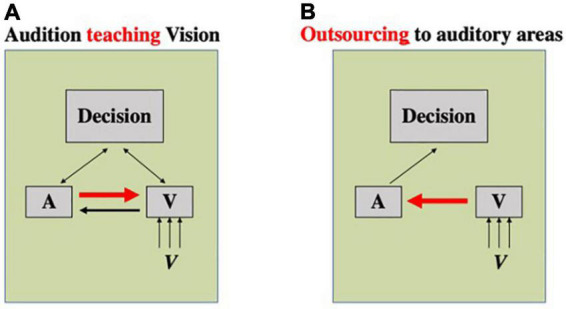
Two possible mechanisms underlying crossmodal transfer of perceptual learning. Boxes A, V, and decision represent, respectively, auditory and visual processing stages, and a decision-making stage of processing. **(A)** A mechanism wherein one sense is able to teach another. In this case, a sense that is superior in performing a task (here, auditory modality) would be able to teach that information to a different sense (here, vision) through crossmodal connections. **(B)** A mechanism wherein one sense outsources the processing to another sense. In this case, a sense that is worse in performing a task (here, vision) will send information to a sensory region that is more apt in processing that task (here, audition) through crossmodal connections.

However, the aforementioned models of multisensory benefit may not be sufficient to account for some existing phenomena. For example, Barakat et al. (2015) study showed that auditory-only training was able to improve visual rhythm discrimination performance similarly to multisensory training. As there was no stimulation of the visual cortex during training, there was no reason that region should be activated sufficiently to surpass the learning threshold to cause learning as would be expected under unisensory theories (Figure 2B). Under multisensory theories, the co-occurrence of the audio and visual signals would be required to change the connectivity between unisensory regions or alter the activation of multisensory regions, and so auditory-only stimulus presentation shouldn’t encourage any changes in the visual modality. Barakat et al. (2015) suggest the possibility of a different sort of multisensory activation: one where the crossmodal connections between sensory cortices can be utilized outside of multisensory training (Figure 3). Under the assumption that there is pre-existing connectivity between sensory regions (e.g., Eckert et al., 2008; Beer et al., 2011) and also between sensory regions and decision regions (e.g., Heekeren et al., 2008; Siegel et al., 2011), this could operate in two ways. It is possible that one sensory region could “teach” another– in the example of Barakat et al. (2015), the auditory region is able to “teach” the visual region (Figure 3A). At test, the visual region is activated, and this will, in turn, cause partial activation of the auditory region, due to their crossmodal connections. Training of the participant in the auditory condition will result in refined processing within the auditory cortex, and activation of this region will allow for signals from auditory cortical regions to help refine the visual processing, improving visual performance. Alternatively, due to crossmodal connections and the putative superiority of the auditory cortex in temporal processing (Glenberg et al., 1989; Repp and Penel, 2002; Mcauley and Henry, 2010; Grahn, 2012), the visual region could outsource processing on this task to the auditory region almost entirely (Figure 3B). Here, activation of the visual region would excite the auditory region through crossmodal pathways and, as the trained auditory region is thus activated sufficiently to be used in the decision-making process for the visual decision. Under either of these models, it is possible for unisensory training in one modality to influence performance in another modality, provided the regions are connected crossmodally or via a multisensory convergence area. Still, it is not clear why multisensory training would not result in a superior outcome to auditory-alone training. Future studies will need to explore the role of relative dominance of the two modalities in a given task as well as other factors such as task difficulty and duration of training to shed light on the underlying mechanisms and the factors that determine the effectiveness of multisensory training relative to unisensory training in a given task for a given individual.

## 5. Discussion and future directions

In the realms of human learning and memory, it has been continually shown that taking advantage of multisensory training/encoding can improve later performance, including performance in unisensory tasks. Exposure to correlated or redundant crossmodal stimuli has been shown to lead to faster learning and enhanced unisensory processing in perceptual learning tasks (as in Kim et al., 2008; Barakat et al., 2015). Similarly, passive exposure to co-occurring sensory input across modalities (resulting in the acquisition of a novel association) can also lead to improved unisensory processing (as in Ernst, 2007; Seitz et al., 2007). Repeated mismatch across the senses can also result in learning via recalibration of sensory representations (as in Wozny and Shams, 2011). Multisensory encoding of stimuli has been shown to improve later recall for visual and auditory stimuli, even when recall cues are unisensory (as in Lehmann and Murray, 2005; Moran et al., 2013; Duarte et al., 2022; Murray et al., 2022). Altogether these results clearly show that the human nervous system is acutely sensitive to the relationship between sensory signals across modalities, and exposure to multisensory stimuli, not only refines multisensory processing (see Quintero et al., 2022; Mathias and von Kriegstein, 2023; for reviews), but it also alters and refines unisensory representations and the ensuing unisensory processing.

While we have posited possible models for the observed improvement above, it should be noted that this is non-exhaustive– several possible mechanisms may be at play, separately or in combination. While Mathias and von Kriegstein (2023) point out that multisensory models capture neuroscientific evidence better, many important questions regarding the neural mechanisms of perceptual learning remain unanswered. For example, it is not clear to what degree and under which conditions the benefits of multisensory training and encoding stem from alterations in crossmodal connectivity versus changes in activity of multisensory regions versus refined representations in unisensory regions. Some recent work in animals even suggests that multimodal experience fundamentally changes the cooperative nature of how senses relate; they claim that the natural interaction of the senses is one of competition, which can be shaped into cooperation through multisensory experience (Yu et al., 2019; Wang et al., 2020). If such cooperative organization is truly only available with multisensory experience, then multisensory learning may reflect an even more complex shift in the relationship between multimodal and unisensory brain regions. It is also not clear under which conditions “unisensory” processing regions (such as visual cortex or auditory cortex) are involved in providing a “teaching signal” to another modality and/or outsource processing to another sensory region. Clarifying which circuits or pathways best capture learning and memory benefits stemming from multisensory exposure should be the focus of future research. Understanding these neural mechanisms would allow us to better understand and harness them for improving human learning and memory performance.

Perhaps an even more important target for further research would be to uncover computational principles governing multisensory learning. While some general ideas have been proposed in the literature there are few attempts to comprehensively and rigorously model how the brain benefits from multisensory stimulus presentation in learning/memory contexts. Rigorous computational modeling is needed to shed light on the nature of information processing involved in the different sensory conditions during learning and provide an understanding of how it is possible to achieve the same level of accuracy in unisensory conditions and multisensory conditions after multisensory training (see the discussion of Kim et al., 2008 in the section “2.1. Perceptual learning”).

With regards to memory, there are many behavioral observations that span decades supporting that multisensory and unisensory information appears to interact in the memory system, yet computational models are lacking. For example, the phenomenon of redintegration (Horowitz and Prytulak, 1969), where unisensory information can cue a memory with information across multiple senses, has been long cited as a behavioral phenomenon, yet the mechanism by which the senses are entangled in memory remain unclear. Mathias and von Kriegstein (2023) review computational approaches to this question and propose that a Predictive Coding framework can account for some of the findings. While this is a good start, future studies should engage in model comparison and aim to offer computational models that can quantitatively account for the empirical findings. Computational models are needed to formalize an understanding of the way sensory cues work in memory, and to make testable predictions about conditions and the nature of crossmodal interactions and presence and type of multisensory benefit in learning across tasks and sensory conditions.

A better mechanistic and computational understanding of the mechanisms behind multisensory learning and memory benefits would also allow for us to better harness these mechanisms and principles to improve memory and learning in everyday life. Multisensory stimulus presentation is often relatively simple to implement, especially with current technologies, and would provide an easy avenue to bolster learning and memory in a number of contexts. As discussed, the above studies of implicit learning have shown that even arbitrary associations can be quickly learned, and subsequently serve as the basis for improved unisensory processing. Therefore, the benefits of multisensory training/encoding are not limited to only naturalistic tasks. Further research into how multisensory benefits could be applied to everyday tasks could provide a useful avenue to improve human cognitive performance in day-to-day life and guide the development of more effective educational and clinical practice.

The recent findings on benefits of multisensory learning as reviewed here and elsewhere (Shams and Seitz, 2008; Mathias and von Kriegstein, 2023) are also noteworthy in that they may warrant a shift in how the fields of neuroscience and psychology view perceptual learning. These findings have generally been framed [including by us in Shams and Seitz (2008)] as superiority of multisensory learning over unisensory learning. However, a more rational framing may be to view them as showing the inferiority of unisensory learning compared to multisensory learning. In other words, it can be argued that the longstanding tradition of studying learning in unisensory settings has biased interpretation of these findings as reflecting a multisensory benefit, as opposed to recognizing a disadvantage in the unisensory protocols. The world around us provides constant crossmodal information– it’s possible the brain would develop to treat this as a “default” level of information available for learning and memory. If the brain is truly developed to utilize multisensory cues when learning about the environment, then providing less information, as in unisensory learning paradigms, could be forcing the system to use impoverished computational resources for learning. This would lead to an inferior outcome for learning compared to when multisensory cues are available and full computational resources would be used. Under this assumption, multisensory perception is the naturalistic baseline for the brain, which unisensory approaches cannot fully explore. Just as we need information across many senses to truly understand our world, we will need to study the dynamic interplay between the senses to truly understand the human mind.

## Author contributions

LS and CM: conceptualization and writing. Both authors contributed to the article and approved the submitted version.
